# Benzoyl peroxide in the treatment of acne: are there potential health concerns?

**DOI:** 10.3389/fped.2025.1599491

**Published:** 2025-07-21

**Authors:** Sonia Czyz, Kerry Yang, Fatemeh Jafarian

**Affiliations:** ^1^ Department of Medicine, Cumming School of Medicine, University of Calgary, Calgary, AB, Canada; ^2^ Division of Dermatology, Department of Medicine, Cumming School of Medicine, University of Calgary, Calgary, AB, Canada

**Keywords:** acne vulgaris, benzoyl peroxide, benzene, pediatric, safety, public health, dermatology, neoplasm

## Abstract

Benzoyl peroxide is a widely used and effective topical treatment for acne vulgaris, particularly in pediatric and adolescent populations. Despite its established safety profile, recent concerns emerged regarding its potential to decompose into benzene, a known carcinogen, under specific environmental conditions, like elevated temperatures and exposure to ultraviolet radiation. In this paper we review the management of acne vulgaris in pediatric patients, examine the evidence supporting benzoyl peroxide use and explore the recent studies evaluating the association between benzoyl peroxide use and malignancy risk. While initial reports raised alarm over benzene formation, subsequent investigations have not demonstrated an increased risk of hematologic malignancies. Here, we assess the strengths and limitations of existing evidence and identify future research priorities. Additionally, we provide recommendations for the safe and evidence-based use of benzoyl peroxide in pediatric acne treatment.

## Introduction

Acne vulgaris is a common inflammatory disorder of the pilosebaceous unit with an estimated prevalence ranging from 26.8% to 96% globally (1). Although acne vulgaris can present in all age groups, the peak incidence occurs during the teen years, affecting up to 85% of adolescents and young adults (2, 3). The inflammatory manifestations of acne can also damage skin, leading to disfiguring scars and pigmentation changes. Consequently, acne vulgaris is often frustrating for pediatric patients and has been linked to mood disorders, social isolation, and impaired health-related quality of life (HRQoL) (4, 5). Given the high burden worldwide, it is necessary to encourage awareness around therapeutics used to address acne in younger populations.

One of the mainstay treatments for acne vulgaris is benzoyl peroxide (BPO). Known for its safety, minimal irritation at low concentrations (2.5%–5.0%), and efficacy in reducing *Cutibacterium acnes*, BPO is widely available in over the counter (OTC) and prescribed formulations. However, recent studies raised safety concerns, suggesting BPO in skin care products can decompensate into benzene, a recognized carcinogen (6). Given the significance of BPO in acne treatment, we sought to investigate this topic further. This perspective synthesizes current evidence on the safety of BPO, including concerns about benzene formation and studies that challenge this risk, and outlines future research directions alongside recommendations for pediatric acne management.

## Acne vulgaris in the pediatric population

Acne lesions can be divided into “non-inflammatory” open and closed comedones, or “inflammatory” papules, pustules, nodules, and cysts. The pathogenesis of acne is multifactorial and traditionally involves a complex interplay of four main factors: (1) excessive sebum production, (2) hyperkeratinization of pilosebaceous follicles, (3) bacterial colonization of sebaceous follicles with *C. acnes* and (4) inflammation. In the initial stages of comedone formation, it is suggested that a hyperkeratotic plug forms and acts as a bottleneck for the accumulation of shed keratin and sebum, which clogs follicles. Androgen hormones and insulin-like growth factor 1 are thought to increase sebum secretion, which may explain why acne emerges during the pre-adolescence phase (7). *C. acnes* colonization can upregulate innate immune responses and prime a pro-inflammatory milieu, that worsens with comedo rupture (8, 9). In nodular or cystic acne formation, there is pronounced inflammation, which can lead to more painful, slow healing lesions.

Acne vulgaris typically first appears between the ages of 12 and 24. When acne develops before puberty, it is categorized into different age groups. Neonatal acne arises within the first 2–3 weeks of life and is relatively common, affecting 20% of newborns (10). It resolves on its own by 3 months of age and is believed to result from an inflammatory response to *Malassezia*, a yeast that is a component of the normal skin microbiome. Infantile acne onsets between 1 and 12 months of age. It tends to be less prevalent, and includes comedomes, papulopustules and nodules seen in classic acne. Interestingly, infantile acne may reflect physiologic elevations of androgen levels in infants and these patients often have a family history of severe acne (10). Mid-childhood acne, occurring between 1 and 7 years of age, is quite rare. Since this age group lacks a physiologic source of androgens, hyperandrogenism should be ruled out as a cause (11). Pre-adolescent acne emerges between 7 and 12 years of age and is suggested to be more common now due to the trend for earlier onset of puberty (12).

## Current therapeutics for acne vulgaris

Mild to moderate acne is commonly managed with topical therapies. First-line treatment is a topical retinoid to reduce sebum production and regulate ductal keratinocyte growth (13). BPO is strongly recommended as it was shown to reduce both inflammatory and noninflammatory acne lesions (14, 15). Topical antibiotics, like erythromycin and clindamycin, are utilized to reduce inflammation and colonization with *C. acnes* (16). However, rising antibiotic resistance has limited the standalone effectiveness and poses broader public health risks (17). To mitigate resistance, BPO is suggested to be used with antibiotics concomitantly (18). Overall, multimodal regimens remain the standard of care (16).

Moderate-to-severe acne can be treated systemically. This involves the use of oral tetracyclines, primarily doxycycline or minocycline. Hormonal agents, like combined oral contraceptives and anti-androgen medications, can be used in individuals that have hormonally responsive acne, but have a multitude of side effects to take into consideration due to its systemic nature. Oral isotretinoin is known for its excellent efficacy in treating severe, treatment refractory nodulocystic acne (19, 20).

For infantile or pre-adolescent acne vulgaris, these conditions are managed with topical antibiotics, BPO and retinoids. Oral tetracyclines may be prescribed for acne in pediatric patients aged 9 years and older. However, caution is warranted as tetracyclines can bind to calcium in developing teeth, causing permanent discoloration (21, 22). If severe, isotretinoin can be used, but it would be off-label as it is not Food and Drug Administration (FDA)-approved for treatment of acne in children under 12 years of age. Moreover, hormonal therapies are not indicated for preadolescent acne.

## Benzoyl peroxide in treating pediatric acne

BPO reduces bacterial colonization of the skin and possesses mild keratolytic effects. Its antimicrobial properties stem from the release of free oxygen radicals that disrupt protein function in cellular membranes (23), reducing the survival of common cutaneous bacteria and yeasts, including *C. acnes*, *Staphylococcus epidermidis*, and *Malassezia* spp (24). As emphasized earlier, antibiotic resistance in *C. acnes* continues to rise with antibiotic use for acne. In contrast, BPO remains effective without reported resistance. Side effects of BPO include burning sensation, dryness, erythema, peeling, irritation, and bleaching of hair and clothes. However, BPO is a dose-dependent skin irritant; therefore, lower-concentration formulations and wash-off product options are better tolerated as observed in patients 12 years and older (14). It is important to note that BPO's bactericidal properties are not concentration dependent (14).

While BPO is widely recommended by both North American and European guidelines (16, 25, 26), the majority of literature focuses on adults and data on pediatric populations are limited (Table 1). Eichenfield and colleagues conducted multiple studies in patients with ages ranging from 10 to 17 years old with acne vulgaris, evaluating the safety and efficacy of BPO in combination with topical retinoids or antibiotics (27–29). Their findings demonstrate that these regimens are generally well tolerated, with minimal adverse events. Consistent results have been observed across additional studies in both preadolescent and adolescent populations, supporting BPO, whether in combination therapy or as monotherapy, as a safe and effective treatment modality in this age group (30–35). Additionally, the skin microbiome of preadolescent females (7–12 years) was profiled following daily 4% benzoyl peroxide wash for 4–8 weeks (36). BPO use was reported to be associated with decreased acne lesions without significant changes in skin microbial diversity (36).

**Table 1:** Summary of studies assessing BPO safety and efficacy in the pediatric population.

Study	Age (years)	Product tested	Safety outcomes	Reported side effects	Efficacy outcomes	Reference
Cook-Bolden, 2015	12–17	Clindamycin/BPO Gel 1.2%/3.75%	Well tolerated. Mean tolerability score <1.	Erythema, scaling, burning/stinging, itching	Significant reduction in inflammatory and noninflammatory lesions by week 12.	(33)
Del Rosso et al. 2023	≥9	Microencapsulated tretinoin/microencapsulated BPO cream 0.1%/3%	Well tolerated. Mean tolerability score <1.	Erythema, scaling, dryness, burning/stinging	A significantly higher percentage of patient achieved IGA by 12 weeks compared to control.	(30)
Eichenfield et al. 2009	11–17	Clindamycin/BPO Gel 1%/5% and three step acne system (2% SA cleanser +2% SA toner +5% benzoyl peroxide gel)	Well tolerated. Mean tolerability score <1.	Erythema, dryness, peeling, burning/stinging, itching	Both formulations were successful in reducing inflammatory and noninflammatory lesions. Three step acne system reduced acne more rapidly.	(27)
Eichenfield et al. 2010	12–17	Adapalene/BPO 0.1%/2.5% Gel	Well tolerated. Mean tolerability score <1	Erythema, dryness, scaling, burning/stinging, itching	Significant reduction in inflammatory and noninflammatory lesions by week 12.	(28)
Eichenfield et al. 2023	10–17	Clindamycin/adapalene/BPO 1.2%/0.15%/3.1% Gel	Relatively well tolerated. One child experienced TEAE.	Erythema, dryness, scaling, burning/stinging, itching	Significant reduction in inflammatory and noninflammatory lesions by week 12.	(29)
Gollnick et al. 2009	12–55	Adapalene/BPO Gel 0.1%/2.5% and BPO 2.5% monotherapy	Well tolerated. Mean tolerability score <1.	Erythema, scaling, dryness, burning/stinging	Significant reduction in inflammatory and noninflammatory lesions by week 12. Combination therapy was superior to monotherapy.	(31)
Kawashima et al. 2017	12–49	BPO 2.5% or 5%	Well tolerated. Mean tolerability score <1.	Skin exfoliation, erythema, nasopharyngitis, WBC increase, pruritus, contact dermatitis	Subgroup analysis of ages 12–18 showed significant reduction in inflammatory lesions at both BPO concentrations at 12 weeks.	(34)
Kawashima et al. 2017	12–49	BPO 2.5% or 5%	Adverse events were mild.	Skin exfoliation, contact dermatitis, erythema, dryness, pruritus	Significant reduction of inflammatory and noninflammatory lesions at 52 weeks.	(32)
Stein Gold et al. 2009	12–17	Adapalene/BPO Gel 0.3%/2.5%	Well tolerated. Mean tolerability score <1.	Nasopharyngitis, skin irritation, eczema	Significant reduction in inflammatory and noninflammatory lesions by week 12.	(35)

BPO, benzoyl peroxide; IGA, investigator's global assessment; SA, salicylic acid; TEAE, treatment-emergent adverse event; WBC, white blood cell.

However, the FDA labelling for various BPO products state the safety and effectiveness in children below the age of 12 have not been established. Definitive evidence to guide optimal BPO management in this age group remains lacking. In practice, clinicians often extrapolate from studies in older populations, leading to the off-label use of BPO in children under 12 years of age. In addition to topical retinoid or antibiotics, there are no absolute contraindications to the use BPO in this population. However, the main concerns include a heightened risk of local irritation. When using off-label treatments in younger children, clinical judgment and close monitoring are essential. A prudent approach includes initiating therapy with the lowest effective concentration and avoiding more irritating formulations, like BPO and retinoid combinations. Strategies to minimize irritation may involve alternate-day or short-contact application during the initiation phase, with gradual titration based on tolerability. Earlier follow up at 2–3 weeks may be appropriate to assess response and tolerance. It is critical to provide the patient and their caregiver education regarding potential side effects and appropriate product use.

When comparing to other treatment regimes, BPO has demonstrated its effectiveness with, typically mild and dose-dependent side effects. Systemic absorption is minimal (37) and allergic reactions are rare. In contrast, while topical retinoids are also first-line agents for comedonal and mixed acne, they may cause more frequent irritation and require additional counseling on sun protection. Topical antibiotics are less preferred as monotherapy due to resistance concerns and should always be combined with BPO. Oral antibiotics are reserved for more severe acne but should be used judiciously due to the systemic impacts.

## Current developments regarding benzoyl peroxide for acne vulgaris

BPO can thermally decompose into benzene, which is a known carcinogen (6). Valisure, an independent testing laboratory based in Connecticut, found that some common OTC BPO products may contain alarmingly high levels of benzene when incubated for days to weeks at elevated temperatures. This was found to be a problem with the intrinsic stability of BPO during delivery or storage of the product rather than an issue with contamination (6, 25). These findings were the reason behind the Citizen Petition filed to the U.S. FDA on March 5th, 2024 (38). In the petition, they called for the recall and suspension of sale of BPO-containing products, along with further investigations and newly updated guidelines around these products (38).

This is particularly relevant since in 2011 the FDA identified BPO as a tumor promoter in animals when combined with a chemical initiator (50). Although animal studies may not reflect human use conditions. Soon after Valisure filed their Citizen Petition, organizations such as the American Academy of Dermatology and the American Acne & Rosacea Society published response statements emphasizing the importance of public health and safety while waiting for further guidance from the FDA (39, 40). Many leading dermatologists published commentaries of their own, calling for more data on BPO testing and transparent independent laboratory verification (41). There were also concerns raised around Valisure's methodology. Although in their study an incubation temperature of 37°C was used to simulate standard body temperature, 50°C to stimulate shelf-life performance at an accelerated stability testing temperature, and 70°C to stimulate transportation/passenger vehicle excursion, BPO products are often stored at room-temperature or are refrigerated.

Over the next several months, various studies using different databases and surveys began publishing findings to help clarify the safety profile of BPO in the real world. Given benzene can induce oxidative damage and genetic and epigenetic modifications in hematopoietic stem cells, hematologic malignancies were a main focus in the BPO safety studies (39). The population studied were 12 years and older.

The first study was done by Veenstra et al. (42). They used Cosmos, which is a dataset created through a community collaboration of health systems on Epic. The study looked at over 2.3 million patients with acne and compared the prevalence of acute myeloid leukemia (AML) in patients with acne with a BPO prescription and those without. Since many patients use OTC BPO products that are not captured in electronic medical records, they also compared AML diagnosis in patients with acne vs. those without acne. The study observed decreased odds of AML in patients with acne with a BPO prescription or reported BPO use, or in patients with acne when compared to the general population to account for potential OTC use, stratified by age (42). The paper was criticized for its methodology due to the primary analysis not properly adjusting for confounders such as age, sex, race and ethnicity, smoking status, and other comorbidities, leading to significant bias. There was also criticism related to the degree of BPO exposure and follow up time, along with the poor methodology used to account for OTC BPO use (43). In response, Veenstra et al. reanalyzed the data after adjusting for age and sex, and did not find any significant differences in odds ratios, but did note that their analysis had several limitations (42).

Although there were issues regarding the Veenstra study, other more robust studies were published during the weeks following that all reported no evidence between BPO use and an increased risk of malignancy. Sadr et al. used the National Health and Nutrition Examination Survey to exact-match 79 individuals who reported BPO use with control individuals who did not use BPO and did not have acne (44). They found no association between BPO use and an increase in the detectable levels of benzene in the blood (44).

Afterwards, there were additional studies using the TriNetX US Collaborative Network, a database with over 132 million patients, to conduct more rigorous analyses. These studies found that patients with acne who received a BPO prescription, as well as patients with acne overall, did not have an increased risk of hematologic malignancies compared to a matched control group (45, 46).

Although these studies had robust statistical approaches, there were several limitations. The degree of exposure was not quantified, meaning there is a significant degree of unaccounted for variability that could have biased the results. Indeed, reported use of BPO among patients likely reflects inconsistencies and a broad range of exposures, from single applications to sustained, long-term use over several years. OTC BPO use was also not accounted for. These studies tried to control for it by comparing patients with acne to a comparator group, such as patients with melanocytic nevi or viral warts, but there are significant limitations with this approach. Firstly, not all patients with acne see a physician to get treatment for it, especially given the current barriers to access. In an online survey, only 25% of teenagers with acne have visited a doctor, with only 52% adhering to their acne treatment (47). This suggests most individuals with acne are categorized incorrectly within these databases. These patients are also more likely to use OTC products to manage their acne, including OTC BPO, meaning there is a significant degree of confounding variables which was not adjusted for. There was also no mention of excluding patients with both acne and the comparator characteristic, which is another potential confounding variable that likely did affect the results. These studies found there was a lower risk of hematologic malignancies with patients coded as having acne within these databases, which is contrary to the expected finding. Also, the risk with benzene depends on cumulative exposure, meaning the risk of adverse health events depends on dose and time. Longer-term follow up in these studies would allow for a better understanding of the risk of benzene exposure in BPO-containing products, along with exploring whether or not BPO increases the risk of other types of malignancies (48).

Valisure more recently published a paper where they tested 111 OTC BPO drug products at room temperature shortly after being acquired off the shelf. Of the 111 BPO products, 38 were above the conditionally restricted FDA limit of 2 ppm for benzene in drug products (49). Levels ranged from 0.16% ± 6% ppm to 35.30% ± 2% ppm, and product age did not seem to affect the levels of benzenes found in the product (49). Stability testing of a prescription encapsulated BPO product revealed no detectable benzene at 2°C (35.6°F), but a high level of formation at 50°C(122°F), suggesting encapsulation may not mitigate thermal degradation, although cold storage may prevent it. Ultraviolet exposure, even at one-third the intensity of peak sunlight, also induced substantial benzene formation (49).

On March 11th 2025, the FDA had announced that they indeed found benzene contamination in a number of BPO acne products, leading to voluntary recalls (46). Reassuringly, more than 90% of tested products had undetectable or extremely low levels of benzene (46). The full results of FDA testing is yet to be published.

## Recommendations for benzoyl peroxide usage

Considering the findings, it is prudent to act cautiously. As research continues to be done regarding the safety of BPO drug products, patient care should continue to be the priority. Moreover, alternative options, such as oral antibiotics or isotretinoin in young patients, have their own risks associated with them (38). While the FDA continues their investigation, the focus should be to minimize any potential risks that might arise from the use of BPO in skin products. This includes educating young patients and their families about safe storage practices, such as keeping products refrigerated, or in an environment not exposed to direct sunlight (49). Instructing patients to move their BPO drug products to a cooler location while showering can also help reduce the risk of benzene formation. Cold chain storage and transportation to retailers or pharmacies needs to be standard practice, and when delivering BPO-containing products, the temperature of the cargo hold should be regularly monitored and upward fluctuations in temperature minimized (43). Batches of products should be regularly inspected throughout the supply chain to ensure benzene levels stay below recommended limits.

Healthcare providers can also counsel pediatric patients on reducing the amount of time BPO spends in contact with the skin and need for sun avoidance and sunscreen to minimize the amount of ultraviolet-initiated benzene formation. Unfortunately, there are no current studies that evaluate if the introduction to benzene-containing products early in life raises unique concerns relative to exposure later in life. With most BPO-containing drug products having acceptable levels of benzene and mounting evidence suggesting that BPO is not associated with increased malignancy risk, both patients and providers need to remain up-to-date regarding BPO's safety profile in acne management and treatment so decisions regarding its use can be made in an evidence-informed manner.

## Conclusion

Acne is a pervasive and often difficult-to-treat condition that commonly affects pediatric and adolescent patients. Thus there exists an imperative to ensure that any future decisions regarding BPO are made in a well-informed and methodical manner. Although the recent findings raise significant concerns around the presence of benzenes in BPO drug products, more research and evidence are needed before any conclusions can be drawn. Research using a variety of different perspectives and methodologies, done in a transparent and rigorous manner, will be crucial to inform next steps regarding the future of BPO. Additionally, many BPO products are marketed to young audiences and prescribed “off-label” to pre-adolescent patients; however, their safety concerning benzene formation has not been studied. As the FDA continues to assess the situation, various measures can be enacted to ensure patient management and safety is prioritized. This includes patient education around proper storage practices, safer transportation and quality assurance measures, and a larger emphasis on risk reduction, such as by explaining to patients the importance of minimizing sunlight exposure when BPO is applied on the skin. It is crucial to remain pragmatic while minimizing any sensationalism that might arise as we wait to see what future steps may be.

## Data Availability

The original contributions presented in the study are included in the article/Supplementary Material, further inquiries can be directed to the corresponding author.
